# Cardiac allergy (Kounis Syndrome) and loss of motor neural sensaton during local anesthetic skin test

**DOI:** 10.1186/1939-4551-8-S1-A210

**Published:** 2015-04-08

**Authors:** Zeynep Ferhan Ozseker, Ismet Bulut, Fatma Merve Tepetam, Bahauddin Colakoglu, Mehmet Emin Kalkan, Ahmet Seyfeddin Gurbuz

**Affiliations:** 1 Sureyyapasa Chest Diseases and Chest Surgery Training Hospital, Turkey; 2 Istanbul University Istanbul Medical Faculty, Turkey; 3 Clinic of Cardiology, Kartal Kosuyolu High Education and Research Hospital, Turkey

## Background

Kounis syndrome is myocardial ischemia following with allergic reactions. It is rarely mentioned in the allergy literature but more than 100 reports in the cardiology. But we didn’t find any article mentioned Konuis syndrome and loss of motor neural sensation during the skin allergy test.

## Methods

31 years old woman was admitted to our Immunology and Allergy outpatient clinic for drug allergy. Her history was; 4 weeks ago she took clindamycin capsule and 4th day she had generalized urticaria. She had dental abscess and dental decay, dentist had been given to her clindamycin and performed local anesthesia for filling her tooth. 5 minutes later she had piruritis in her body, dyspnea, numbness on her tongue and neck. And she had been admitted to the emergency room.

We had to find local anesthetic because she has severe dental ache and cotton plug in her tooth. Prick test was performed with 1/1 dilution and after intradermal test was performed with 1/1000 dilution with lidocaine and articaine. They were negative and there wasn’t any systemic reaction. As soon as intradermal test was performed with 1/100 dilution she had nausea and severe left side chest, neck and back pain, dyspnea and left arm numbness. We administered anaphylaxis treatment (adrenalin 0.3 mg i.m., O2 5 lt/dk, rapid istonik saline serum infusion, feniramin maleat i.v, metylprednisolone 40 mg. i.v.). 5 minutes later urticarial hives were appeared her neck and face, stridor was heard. 0.3 mg adrenalin was injected about seven minutes later and five minutes later then she respiratory arrested. 0.3 mg adrenalin was administered intravenously and she recovered. But her chest pain progressed and left side of her body loosed of motor sensation. We analyzed her serum for cardiac enzymes three hours later and troponin was 2.9 ng/mL, CK-MB: 10.2 U/L. Myocardial infarction was diagnosed and she was transferred to the cardiology intensive care unit. Five hours later her enzymes elevated, 5.81 ng/mL,187 U/L, respectively.

## Results

Coronary angiography was done and it was completely normal. About 24 hours later she recovered and all of her symptoms were disappeared. She discharged from hospital.

## Conclusions

Kounis syndrome is rare but it’s life threaten. Almost all drugs cause this syndrome. Drug skin tests may be thinking as safe, but this case show that if there is a suspicion of this syndrome must to be careful. Come into mind and quick treatments this situation may be life saving.

## Consent

Written informed consent was obtained from the patient for publication of this abstract and any accompanying images. A copy of the written consent is available for review by the Editor of this journal.

